# Chinese patients with 3M syndrome: clinical manifestations and two novel pathogenic variants

**DOI:** 10.3389/fgene.2023.1164936

**Published:** 2023-08-31

**Authors:** Ningan Xu, Kangxiang Liu, Yongjia Yang, Xiaoming Li, Yan Zhong

**Affiliations:** ^1^ Department of Child Healthcare, Hunan Children’s Hospital, University of South China, Changsha, Hunan, China; ^2^ The Laboratory of Developmental and Behavioral Pediatrics, Hunan Children’s Hospital Changsha, University of South China, Changsha, Hunan, China; ^3^ The Laboratory of Genetics and Metabolism, Hunan Children’s Research Institute (HCRI), Hunan Children’s Hospital, University of South China, Changsha, China; ^4^ Department of Radiology, Hunan Children’s Hospital, University of South China, Changsha, Hunan, China

**Keywords:** 3-M syndrome, growth hormone, *CUL-7*, *OBSL1*, short stature

## Abstract

**Background:** 3M syndrome is a rare autosomal recessive disease, characterized by intrauterine and postnatal growth retardation, facial dysmorphism, large head circumference, and skeletal changes, has rarely been reported in the Chinese population.

**Methods:** We describe the clinical manifestations and gene variants in four sporadic cases of 3M syndrome in Chinese individuals from different families.

**Results:** All cases had significant growth retardation, relative macrocephaly, and typical facial features. Exome sequencing revealed that two patients with 3M syndrome had homozygous variants of the *CUL7* gene: one novel pathogenic variant and one previously reported pathogenic variant; the other two patients were heterozygous for variants in *OBSL1*, one of which had not been reported previously. Clinical evaluation indicated that these Chinese patients with 3M syndrome shared similar recognizable features with those reported in patients of other ethnic backgrounds, but not all patients with 3M syndrome in this study had normal development milestones. Two patients underwent recombinant human growth hormone (rhGH) therapy and showed accelerated growth in the first 2 years; however, the growth rate slowed in the third year in one case. There were no obvious adverse reactions during rhGH treatment.

**Conclusion:** We report one novel *CUL7* and one novel *OBSL1* mutation in patients with 3M syndrome. Children with short stature, specific facial features, and physical symptoms should be referred for genetic testing to obtain precise diagnosis and appropriate treatment. The effects of rhGH treatment on adult height requires long-term observation and study in a large sample.

## 1 Introduction

3M syndrome (OMIM #273750) is a rare autosomal recessive disease with unknown prevalence, characterized by intrauterine and postnatal growth retardation [≤4 standard deviation score (SDS)], facial dysmorphism, large head circumference, and skeletal changes, with normal intelligence and endocrine function (Al-Dosari et al., 2012; Essaddam and Saayda, 2019); the condition was first reported by Miller, McKusick, and Malvauxx in 1975 and named 3M syndrome (Miller et al., 1975). According to Nosology and Classification of Genetic Disorders of the Skeleton 2015 Revision, 3M syndrome is grouped under “Slender bone dysplasia group” (Bonafe et al., 2015). The main skeletal features are long, slender tubular bones, reduced anteroposterior diameter of the vertebral bodies (tall vertebral bodies), and delayed bone age. Previously, diagnosis of 3M syndrome depended primarily on clinical manifestations and radiographic examination. With the development of gene diagnosis technology, variants in the *CUL7*, *OBSL1*, and *CCDC8* genes have been identified as responsible for 3M syndrome (Guo et al., 2020). In 2005, Huber et al. (2019) initially reported that *CUL7* variants accounted for 77.5%, *OBSL1* variants 16.3%, and *CCDC8* variants 6%, of 3M syndrome cases; however, with continued research, changes in *CUL7* were found to be responsible for the majority of cases, while *OBSL1* mutations were present in more than 16.3% and those in *CDCC8* less than 6%. In one study of 24 patients with 3M syndrome in Turkey, no known causative gene was detected on genetic testing in four patients who met the clinical diagnosis of 3M syndrome, suggesting that there may be other unknown pathogenic genes that contribute to 3M syndrome (Simsek-Kiper et al., 2019; Khachnaoui-Zaafrane et al., 2022).

The *CUL7* gene contains 26 exons and encodes a CUL7 protein containing 16,098 amino acid residues, which has an important role in the synthesis of E3 ubiquitin ligase complexes with SKPl (S-phase kinase-associated protein 1), FBX29 (F-box protein), and ROC1(Regulator of cullins 1). Functional experiments to verify R1445X and H1464P in CUL7 mutations showed that proteins with these two mutations cannot recruit ROC1 to form a complex, indicating that loss of ubiquitination may be the main pathological mechanism underlying 3M syndrome (Flex et al., 2013). In addition, researchers have found that mitotic and cytokinesis defects caused by loss of CUL7 function may be associated with short stature in patients with 3M syndrome (Li et al., 2014).

*OBSL1* consists of 23 coding exons that can generate three spliced forms, encoding proteins that including several immunoglobulin-like domains and a single fibronectin domain, including a protein of 1896 amino acids that is homologous with obscurin, interacts with proteins that anchor myosin filaments, and may be involved in stabilization of the cytoskeletal network (Geisler et al., 2007). *OBSL1* missense mutations are associated with a significant increase in insulin-like growth factor binding protein 5 (*IGFBP5*) mRNA (Huber et al., 2010) and mouse models suggest that IGFBP overexpression inhibits the effects of insulin-like growth factor (IGF) and is associated with growth retardation. Furthermore, although the relationship between OBSL1 and CUL7 is unclear, loss of OBSL1 leads to CUL7 downregulation, implying a role for OBSL1 in maintaining CUL7 protein levels (Litterman et al., 2011).

*CDCC8* is a relatively new evolutionary gene considered to be related to the occurrence and development of malignant tumors, HIV, 3M syndrome, and schizophrenia. In 2011, Hanson et al. first discovered that mutations in *CCDC8* can cause 3M syndrome (Hanson et al., 2011); when the CCDC8 protein was knocked out, there was no effect on CUL7 and OBSL1 protein expression levels, but there was an influence on the centrosome localization of CUL7 in mitotic cells, indicating that CCDC8 can regulate CUL7 localization (Yan et al., 2014). In 2019, Wang et al. (2019) found that embryos from *CCDC8* knockout mice also had intrauterine growth retardation; most embryos died *in utero*, while a few died within 7 days of birth. The cause of intrauterine growth retardation and death was related to a disorder of placental vascular development. The placental developmental disorder, intrauterine growth retardation, intrauterine death, and perinatal death in CCDC8 knockout mice is very similar to that observed in CUL7 knockout mouse embryos; however, the proliferation rate of fibroblasts in CUL7 knockout mouse embryos is low and they exhibit advanced senescence, while the proliferation state of fibroblasts in CCDC8 knockout mouse embryos is the same as that of wild-type cells. These findings suggest that CCDC8 is largely functionally similar to CUL7, but that CUL7 has other unique and independent functions.

Management of short stature is a struggle in these patients and final height can be 5–6 SDS below the mean. GH therapy has been tried on many of these patients with variable success (Huber et al., 2011; Clayton et al., 2012; Meazza et al., 2013). Though generally thought to be ineffective, a positive response was noted in a patient with CUL7 mutation in 2015 (Deeb et al., 2015). One 3M patient was treated with rhIGF1, and the result showed this therapy with minimal height benefit but accelerated weight gain (Yang and Patni, 2020). To date, no more than 200 cases of 3M syndrome have been reported worldwide, and there have only been nine publications reporting 15 patients with 3M syndrome in China. Here, we summarize the clinical features and auxiliary examination results of four patients with 3M syndrome from four Chinese families, and report one novel variant each in *CUL7* and *OBSL1*, expanding the molecular spectrum of the syndrome. The purpose of this study is to improve awareness of rare syndromes and to better understand specific genetic diseases in small for gestational age (SGA) children. Our findings will help to improve the accurate diagnosis and inform clinical decisions on the efficacy of growth hormone treatment.

## 2 Materials and methods

### 2.1 Subjects

The study protocol was approved by the Academic Committee of Hunan Children’s Hospital (Approval No. HCHLL-2021-18, Changsha City, Hunan Province, China). Twelve family members (Han Chinese ethnicity), including four children and their parents, provided written informed consent to participate in this study. Parental permission for publishing patient photos was obtained, except for Case 1. Clinical data related to the children were collected through the electronic medical record system, including age at first diagnosis, clinical manifestations, growth and development history, maternal history of pregnancy and childbirth, physical examination, laboratory test results, gene sequencing results, treatment, and prognosis.

### 2.2 Endocrine laboratory tests

Insulin-like growth factor 1 (IGF-1) and IGF-binding protein 3 (IGFBP-3) levels were tested using an IMMULITE 2000 XPi immunoassay analyzer (Siemens, Munich, Germany). Insulin, thyroid function, growth hormone stimulation, and adreno-cortico-tropic-hormone (ACTH) tests were performed using a Roche electrochemiluminescence immunoassay system (Roche, Basel, Switzerland).

### 2.3 Radiological examination

Bone age (BA), and pelvic and spinal images were obtained using a Kangda Direct Digital Radiography System, and magnetic resonance imaging (MRI) performed using a Siemens 1.5T system; slice thickness, 5 mm and slice spacing, 0.1 mm. Spin echo sequence was detected in the sagittal and coronal planes, and proton density weighted processing, T2WI weighted processing and T1WI weighted processing conducted. After scanning, imaging data were read and analyzed by the same two film readers. BA was measured by Tanner and Whitehouse 3 (TW3) method.

### 2.4 Genetic testing

DNA was isolated from peripheral blood using a DNA Isolation Kit (Blood DNA Kit V2, CW2553), in accordance with a standard method as we used before (Shen et al., 2022). Genomic DNA was evaluated and quantified using a Qubit fluorometer. DNA samples from each patient were fragmented into 180–280 bp segments using a Covaris bath sonicator. Libraries were prepared and captured using a KAPA Library Preparation Kit (Kapa Biosystems, KR0453), following the manufacturer’s instructions. Quality-passed libraries were sequenced on the Illumina Novaseq 6,000 platform (Illumina, Inc., United States). Raw BCL files were converted into FASTQ files, with 12 Gb of sequence obtained for each sample (average yield, approximately 16.3 Gb; error rate <0.1%). Variants were annotated and filtered by Ingenuity Pathway Analysis (https://variants.ingenuity.com). Common variants (frequency >0.5% in the ExAC, ESP, or 1000G databases) were excluded. Variants were classified following the American College of Medical Genetics and Genomics (ACMG)/Association for Molecular Pathology standards and guidelines (Richards et al., 2015), the Rules for combining criteria to classify sequence variants are shown in Table 1. We can All putative pathogenic variants detected in the patients by whole exome sequencing were confirmed by Sanger sequencing. Parents of probands were also checked by segregation analysis.

**TABLE 1:** Rules for combining criteria to classify sequence variants.

Pathogenic	(i) 1 Very strong (PVS1) *AND*
	(a) ≥1 Strong (PS1-PS4)*OR*
	(b) z2 Moderate (PM1-PM6) *OR*
	(c) 1 Moderate (PM1-PM6) and 1 supporting (PP1-PP5) *OR*
	(d) ≥2 Supporting (PP1-PP5)
	(ii) ≥2 Strong (PS1-PS4) *OR*
	(iii)1 Strong (PS1-PS4) *AND*
	(a) ≥3 Moderate (PM1-PM6) *OR*
	(b) 2 Moderate (PM1-PM6) *AND* ≥2 supporting (PP1-PP5) *OR*
	(c) 1 Moderate (PM1-PM6) *AND* ≥4supporting (PP1–PP5)
Likely pathogenic	(i) 1 Very strong (PVS1) *AND* 1 moderate (PM1–PM6) *OR*
	(ii) 1 Strong (PS1-PS4) *AND* 1-2 moderate (PM1-PM6) *OR*
	(iii) 1 Strong (PS1-PS4) *AND* ≥2 supporting (PP1-PP5) *OR*
	(iv) ≥3 Moderate (PM1-PM6) *OR*
	(v) 2 Moderâte (PM1–PM6) *AND* ≥2 supporting (PP1-PP5) *OR*
	(vi) 1 Moderaté (PM1–PM6) *AND* ≥4 supporting (PP1-PP5)
Benign	i) 1 Stand-alone (BA1) *OR*
	(ii) ≥2 Strong (BS1-BS4)
Likely benign	(i) 1 Strong (BS1-BS4) and 1 supporting (BP1–BP7) *OR*
	(ii) ≥2 Supporting (BP1-BP7)
Uncertain significance	(i) Other criteria shown above are not met *OR*
	(ii) the criteria for benign and pathogenic are contradictory

## 3 Results

### 3.1 Clinical information

**FIGURE 1:**
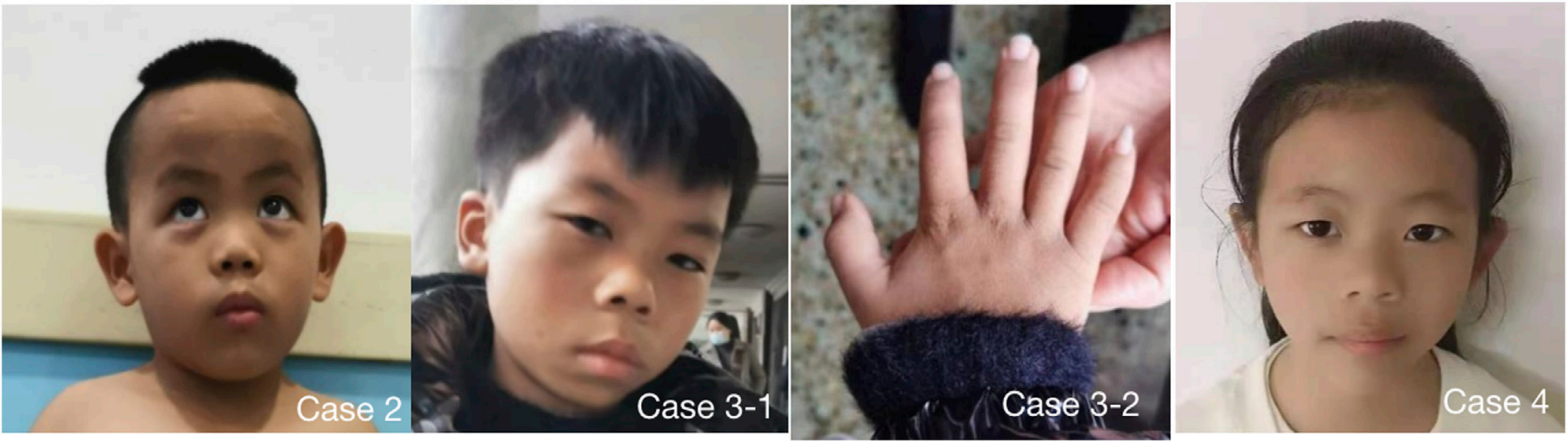
4 cases showed obvious special facial features. Case 2 had triangular face, wide forehead, pointed chin, low bridge of nose, lower eyelid fat pad, lateral canthus oblique, thick lips and thick eyebrows; Case 3 had triangular face, wide forehead, pointed chin, low nasal bridge, thick lips, thick eyebrows and flexion of the fifth finger of right hand (Case 3-2), Case 4 had triangular face, wide forehead, pointed chin, low nasal bridge and thick lips. Case 1 refused to publish portrait photos.

Among the four cases included in this study, two were male and two were female, and their age at diagnos is ranged from 19 months to 11 years 4 months. All four children had obvious short stature (height ≤2 SD), relatively enlarged head circumference, normal intelligence, and particular facial features, such as triangular face, wide forehead, low nose bridge, and thick lips (see Tables 2, 3; Figure 1 for details). Compared with the two children with *OBSL1* gene mutations (116.1 cm, −4.3SD and 132.4 cm, −2.5SD), the two with *CUL7* gene mutations had more severe short stature (90.4 cm, −5.1SD and 96.0 cm, −4.1SD).

**TABLE 2:** Demographic characteristics and genetic variants in four patients diagnosed with 3-M syndrome.

Patient ID	Case 1	Case 2	Case 3	Case 4
Sex	Female	Male	Male	Female
Age of first visit (Years)	0 (born)	5.3	11.3	10.7
Age at diagnosis (Years)	1.4	5.3	11.3	10.7
Current age (Years)	6.9	6.8	11.3	11.0
Gene	*CUL7*	*CUL7*	*OBSL1*	*OBSL1*
DNA variant	c.4969C>T	c.967_993delins	c.1118G>A^#^	c.690dupC
			c.458dupG^∎^	c.458dupG
Protein variant	Arg1657*; EX25/C25	S323Qfs*33	p.W373*	p.E231Rfs*23
			p.L154Pfs*100	p.L154Pfs*100
Novel or reported	Reported	Novel	∎Reported	Reported
			#Novel	
ACMG classification	Pathogenic	Pathogenic	Pathogenic	Pathogenic

*: Means translation termination, #: indicates the variant of “c.1118G>A”, ∎: indicates the variant of “c.458dupG”.

**TABLE 3:** Clinical features of four patients diagnosed with 3-M syndrome.

Patient ID	Case 1	Case 2	Case 3	Case 4
Weeks of gestation	41^+2^	38	40	39^+3^
Delivery mode	Vaginal delivery	Vaginal delivery	Cesarean section	Cesarean section
Abnormal findings in prenatal examination	IUGR	IUGR	IUGR	IUGR
Birth length (cm)	43.0	38.0	41.0	NA
Birth weight (g)	2850	2150	2700	2300
Birth head circumference (cm)	34.0	33.0	NA	NA
Current height (cm)	90.4	96.0	116.1	132.4
Current height, SDS	−5.1	−4.1	−4.3	−2.5
Current weight (kg)	13.05	21.1	24.0	40.5
Current head circumference (cm)	50.5	51.3	53.5	54.0
Growth retardation	+	+	+	+
Normal intelligence	+	+	−	+
Relative macrocephaly	+	+	+	+
Facial features				
Triangular face	+	+	+	+
Frontal bossing	+	+	+	+
Pointed chin	+	+	+	+
Low nasal bridge	+	+	+	+
Presence of lower eyelid fat pads	−	+	−	−
Down-slanted palpebral fissure	−	+	−	−
Full lips	+	+	+	+
Prominent eyebrows	+	+	+	−
Musculoskeletal features				
hyperextensible joint	−	−	−	−
Tall vertebral body	−	+	NA	NA
Lumbar hyperlordosis	−	+	−	−
Small pelvis	−	+	+	+
long slender bones	−	+	+	+
fifth finger flexed	−	−	+	−
Delayed bone age	+	−	+	−

IUGR, Intrauterine growth restriction; NA, no information obtained; +, Positive phenotype; −: negtive phenotype.

### 3.2 Laboratory examination

Routine blood and urine tests, liver and kidney function, electrolytes, blood glucose, and blood lipids were in the normal range in all cases, as were thyroid function, ACTH, and cortisol. IGF-1 and IGFBP-3 levels were also in the normal range. One patient had a peak value of 5–10 ng/mL in the growth hormone stimulation test, while the other three children had peak values >10 ng/mL (see Table 4 for details).

**TABLE 4:** Growth hormone stimulation test results and treatment information.

Patient ID	Case 1	Case 2	Case 3	Case 4
Basal IGF-1 (ng/mL)	60.4	224	197	98.2
Basal IGFBP-3 (µg/mL)	2.8	4.54	4.40	3.54
Basal GH (ng/mL)	0.59	0.85	1.05	0.350
GH peak (ng/mL)	12.99	11.18	18.04	6.62
Baseline height, SDS	−5.1	−4.1	−4.3	−2.5
Baseline annual growth rate (cm/year)	4.4	4.5	4.5	5.0
GH initial dose (IU/kg/day)	0.15	NA	NA	0.12
GH current dose (IU/kg/day)	0.18			0.18
Height ∆SDS/year following GH therapy	0.3	NA	NA	0.4/0.4/0.1
Annual growth rate following GH therapy (cm/year)	5.8	NA	NA	8.0/6.5/5.0

Abbreviation: SDS, standard deviation score.

### 3.3 Imaging examination

**FIGURE 2:**
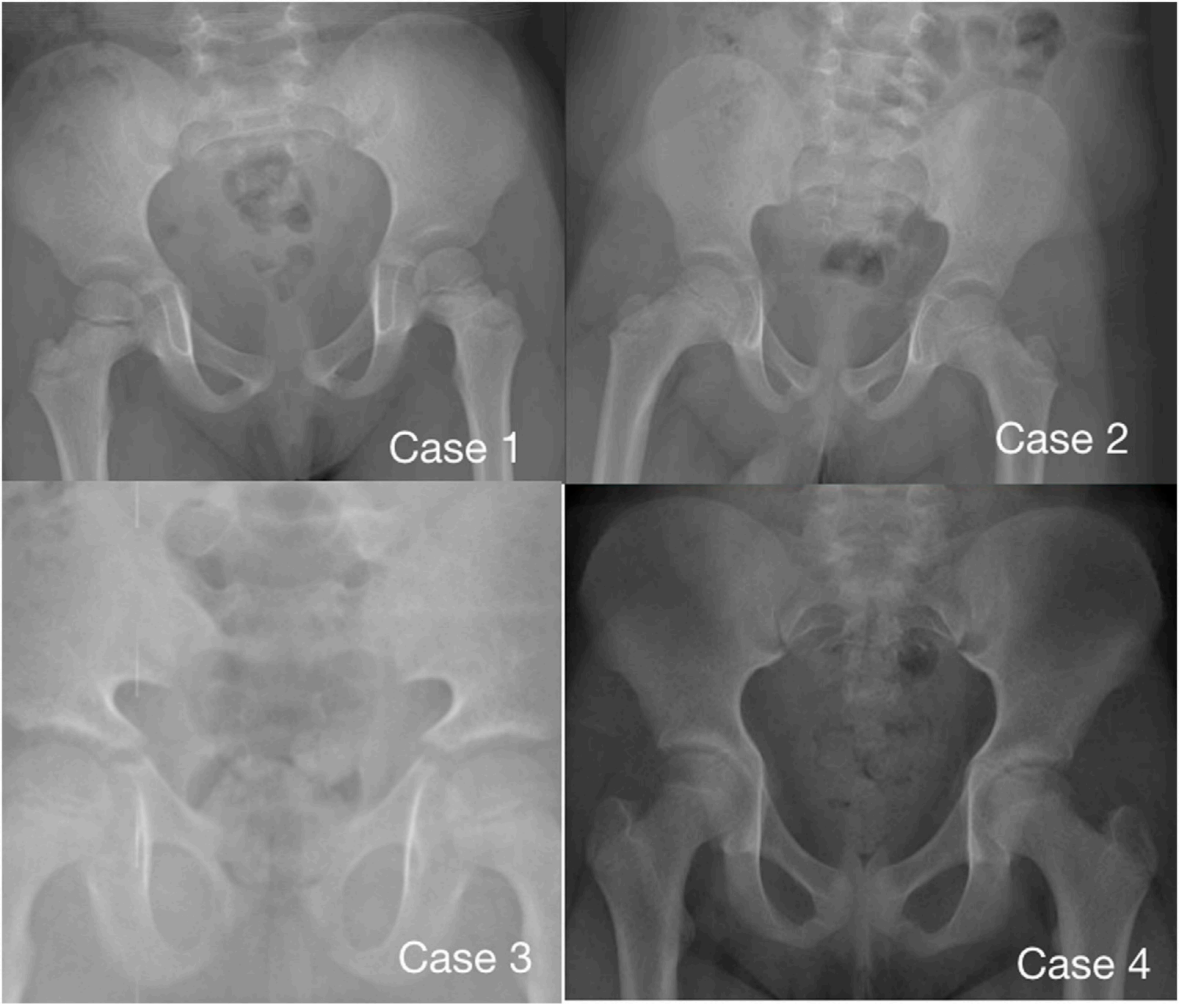
Pelvic radiographs of 4 children with 3M syndrome. No obvious abnormality was found in Case 1. The pelvic measurements of Cases 2, 3 and 4 are smaller than those of their peers. Case 3 shows a typical triangular pelvis.

**FIGURE 3:**
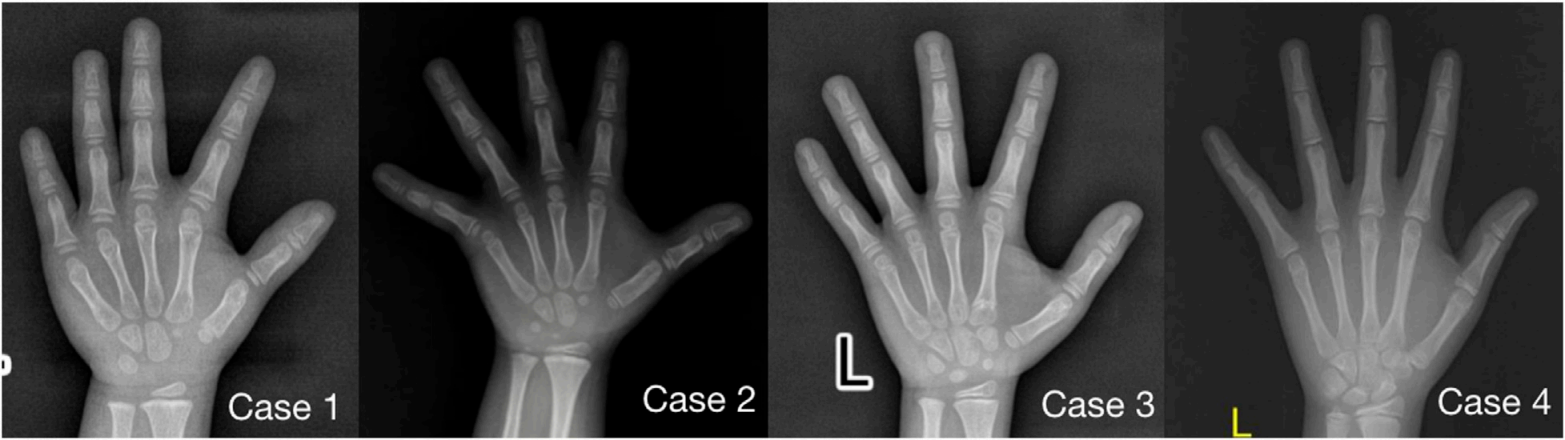
Anterior-posterior radiographs of the metacarpal and phalangeal bones of four children with 3M syndrome. The picture of Case 1 was taken at 5.3 years old, without obvious slender changes of tubular bone, and the BA was 4.0 years old; Case 2 was taken at the age of 5.3 years old, with slender tubular bone and BA basically consistent with CA; Case 3 was taken at 11.3 years old, the tubular bone was slender and the BA was 8.5 years old, significantly younger than CA; Case 4 was taken at 10.7 years old, with slender tubular bone and BA was 11.9 years old, significantly elder than CA.

**FIGURE 4:**
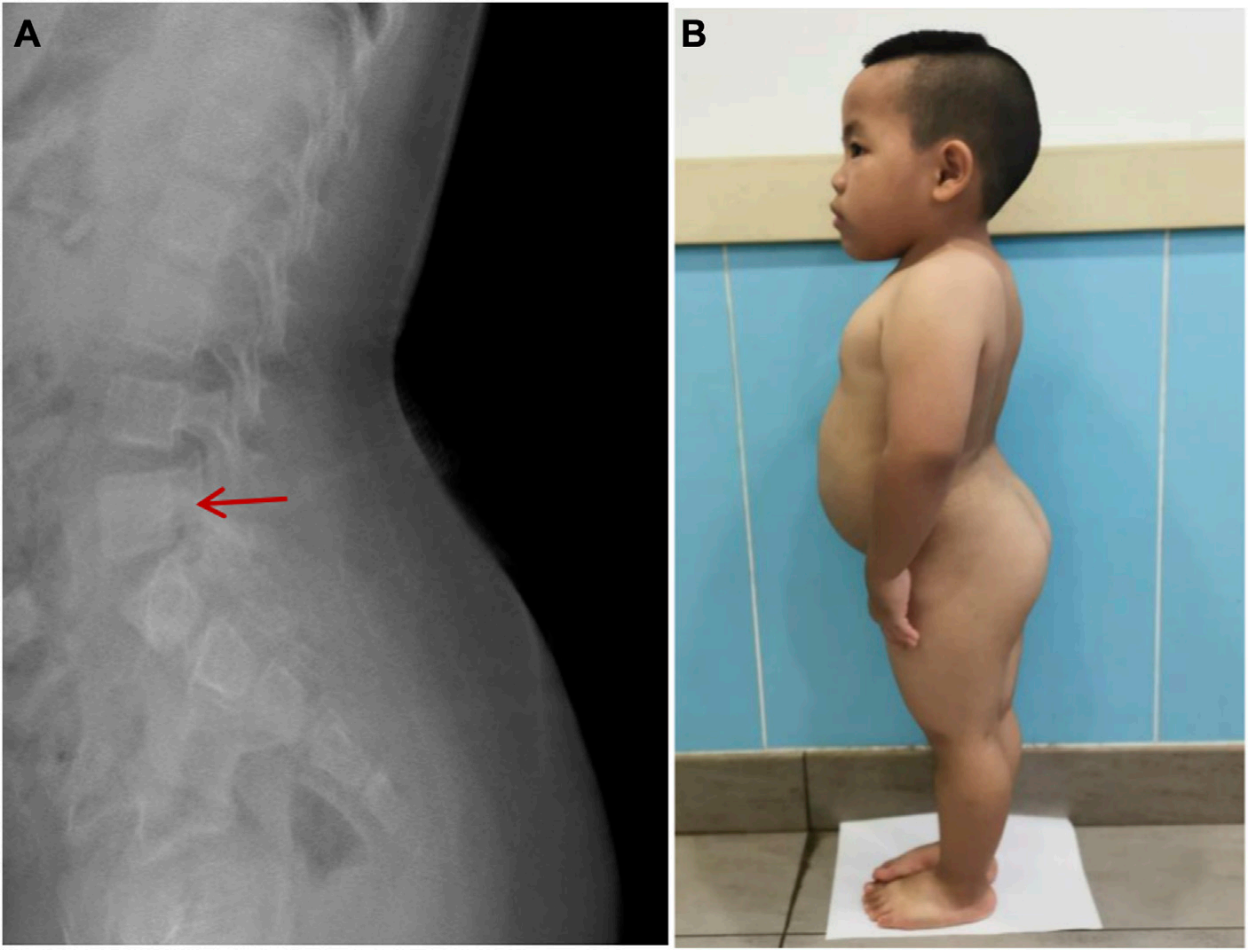
High vertebral body and lumbar hyperlordosis changes in Case 2.**(A)**: The typical high vertebral body and lumbar hyperlordosis (red arrow). **(B)**: Case 2 showed the special posture of waist and buttocks.

Imaging examination showed that three patients had a small pelvis (Figure 2) and long tubular bones (Figure 3), and one patient had typical tall vertebral body of 3M syndrome (Figure 4). Two patients showed BA was significantly younger than chronological age (CA) (Case 1: BA 4.0/CA 5.3; Case 3: BA8.7/CA11.3), and one patient showed advanced bone age (Case 4: BA 11.9/CA10.7). No abnormality was found on MRI examination of the head and pituitary glands of any patient.

### 3.4 Genetic testing

**FIGURE 5:**
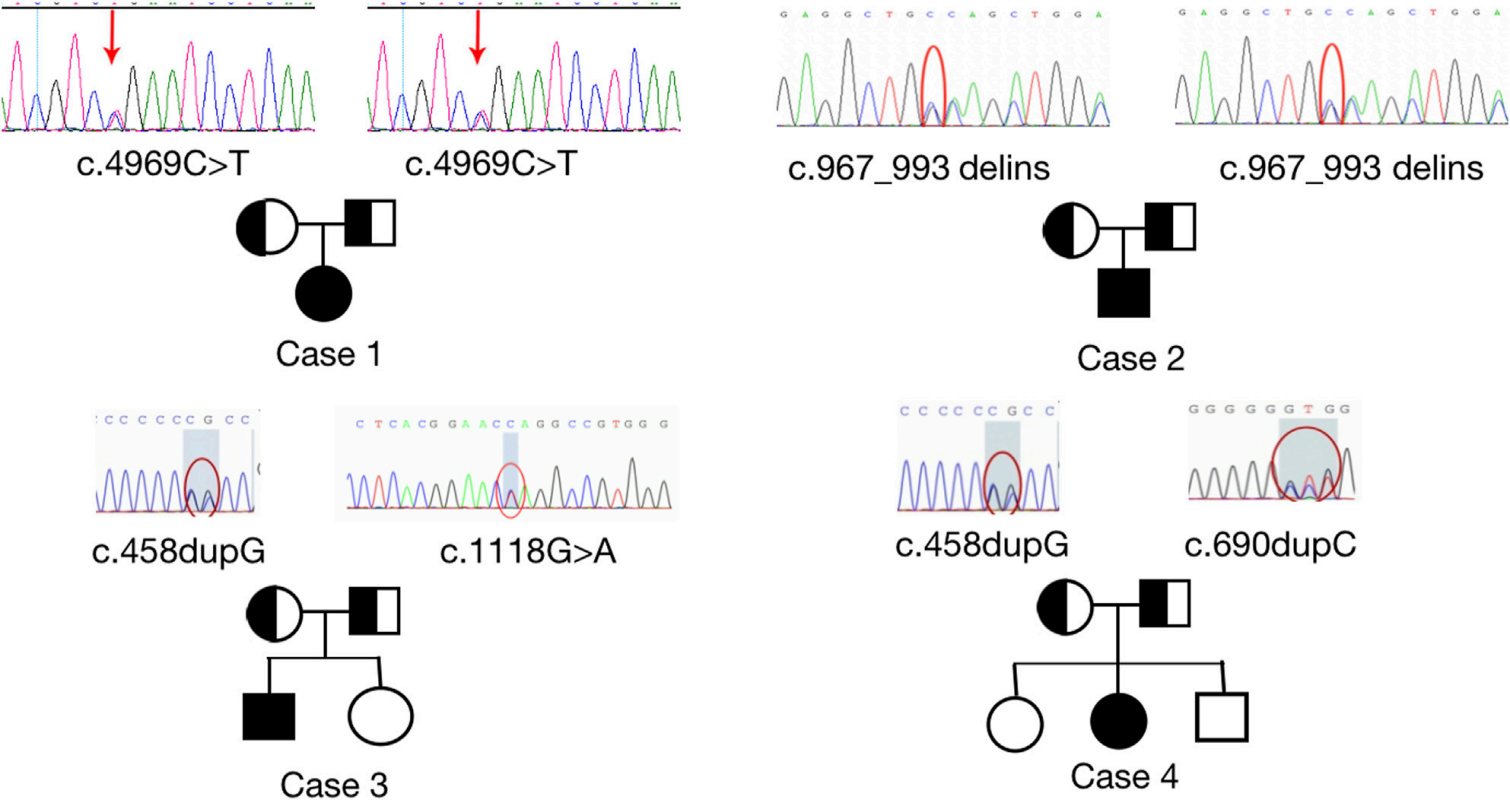
Sanger sequencing results to confirm pathogenic variants and their origin in each family. Homozygous variants, c.4969C>T; p. (Arg 1,657*) and c.967_993delinsCAGCTGG; p. (S323Qfs*33) were identified in *CUL7* (upper images). Heterozygous variants, c.1118 G>A (p.W373*), c.690dupC (p.E231Rfs*23), and c.458dupG (p.L154Pfs*100) were identified in *OBSL1*. Sanger sequencing confirmed that the variants were inherited from the parents of the patients with 3M syndrome.

All four patients were sporadic and five mutations were identified by whole exome sequencing, all of which were verified by Sanger sequencing (Figure 5). Case 1 had a homozygous variant, c.4717C>T (p.R1573*) in exon 25 of *CUL7*, which has been reported previously (Xuyun et al., 2017) and was adjudicated as a pathogenic variant (PVS1+PM2+PM3) according to the ACMG guidelines. In Case 2, a homozygous variant c.967_993delinsCAGCTGG was found in exon 4 of *CUL7* gene. The variant causes a frameshift in the reading frame, resulting in the mutation of the 323rd amino acid of the *CUL7*-encoded protein from serine to L-Glutamine and an premature stop codon (p.S323Qfs * 33). Given that such variant occurred nearing to the N-terminal of the protein, it may cause nonsense-mediated decay at mRNA level. Then the variant fulfill the criteria of null variant (PVS1). The is absent in the public exome database (PM2). The patient’s clinical phenotype is in consistent to 3M syndrome, and 3M syndrome is a rare disorder (PP4). According to the ACMG genetic variation classification criteria and guidelines, it was determined that the variant c.967_993delinsCAGCTGG was pathogenic (PVS1 + PM2 + PP4). Case 3 carried compound heterozygous variants in exon 1 (c.458dupG; p. L154Pfs*100) and exon 2 (c.1118G>A; P.W373*) of *OBSL1*, where the c.1118G>A variant has not previously been reported in the literature. The mutation causes a change in base 1,118 from G to A, leading to premature stop codon, resulting in protein truncation (PVS1), which is very rare in population data and not a polymorphism (PM2); the patient phenotype was highly and specifically consistent with 3M disease (PP4) and therefore c.1118G>A was classified as a pathogenic variant (PVS1 + PM2 + PP4) according to the ACMG guidelines. *OBSL* gene encodes a protein which contains 1896 amino acids. The c.1118G>A variant was a truncation variant from the N-teminal (373*). Such novel variant was high likely resulting in a haploinsufficienty which as the consequence of non-sense mediated decay. The c.458dupG variant was previously documented (Hanson et al., 2009) and can be classified as a pathogenic variant according to the ACMG guidelines (PVS1 + PM2 + PM3 + PP4). Two compound heterozygous variants, c.690dupC (p.E231Rfs*23) and c.458dupG (p.L154Pfs*100), in exon 1 of *OBSL1*, were detected in Case 4. Both variants were previously reported in the literature (Zhang et al., 2019), and are classified as pathogenic variants (PVS1 + PM2 + PP4) according to the ACMG guidelines.

### 3.5 Treatment with recombinant human growth hormone (rhGH) and follow-up

Case 1 started to receive rhGH injection at 4 years old, with an initial dose of 0.15 U/(kg/day), which was gradually increased to 0.18 U/(kg/d). No discomfort occurred during the treatment. The patient was followed up for 34 months, and the height increased by 19 cm and weight increased by 6.05 kg; rhGH treatment has continued until the present time. Case 4 started to receive rhGH treatment at 8 years old, at an initial dose of 0.12 U/(kg/d) and gradually increased to 0.18 U/(kg/d). No discomfort occurred during treatment, and the height increased by 8.0 cm and 6.5 cm in the first and second years, respectively; however, the rate of increase in height slowed significantly in the third year of treatment and the parents withdrew rhGH treatment.

## 4 Discussion

3M syndrome is a rare hereditary disease characterized by intrauterine and postnatal growth retardation, abnormal facial features, enlarged head circumference, skeletal changes, and normal general intelligence and endocrine function, which has rarely been reported in the Chinese population, with less than 20 cases published to date (Xuyun et al., 2017; Zhang et al., 2019; Huang et al., 2021). No *CDCC8* gene mutations have been found in Chinese patients. Compared with the results reported from countries other than China, the proportion of patients with 3M syndrome and mutations of the *OBSL1* gene is higher in the Chinese population, while the proportion of those with *CDCC8* mutations is lower (Huber et al., 2019; Simsek-Kiper et al., 2019). Short stature is the main clinical manifestation of 3M syndrome. It was reported that the height of patients with *CUL7* mutations is significantly lower than that of patients with *OBSL1* mutations (Hanson et al., 2009; Khachnaoui-Zaafrane et al., 2022). In this study, the SDS values of the two children with *CUL7* mutations were lower than those with *OBSL* mutations; however, whether there is a significant difference in height between patients with different genotypes needs further investigation in a larger sample size. Notably, most of the reported 3M syndrome presented with severe dwarfism (−4∼6 SD), but the height of Case 4 remained at the level of −3 SDS before treatment and increased to −2.5 SDS after rhGH therapy. The diagnosis of 3M syndrome has been confirmed by genetic testing until rhGH treatment was unsuccessful. This gives us two hints: firstly, not all of the 3M syndrome patients are severely short, and some clinicians do not understand the clinical manifestations of this rare disease, so there may be missed or misdiagnosed patients based on China’s huge population. Secondly, when SGA patients are not responding well to rhGH therapy, or have specific clinical manifestations, such as specific facial features, skeletal changes or advanced bone age, genetic testing should be performed.

There have been no previous reports of obesity in 3M patients in other ethnicities. Ming Yang and Nivedita Patni reported that a Hispanic male 3M patient developed obesity after treatment with rhIGF1, and BMI decreased after stopping rhIGF1 (Yang and Patni, 2020). In this study, the BMI of Case2 has reached 22.89 kg/m^2^ (>99th percentile) and over the level of severe obesity. Whether Chinese 3M patients are more likely to develop obesity and metabolic syndrome remains to be studied in larger samples.

Patients with 3M syndrome are considered to undergo normal sexual development (Beyhan et al., 2021). We noticed that puberty initiated at the expected time in Case 4, who carried an *OBSL1* gene mutation, while bone age was accelerated. Kyung Lee et al. (2020) reported a pair of sisters with 3M syndrome with central precocious puberty, who also had *OBSL1* mutations. Whether precocious puberty or rapid puberty progression is a particular manifestation associated with changes in *OBSL1* mutation or a phenotype more common in Asians, further clinical observation is needed. The presence of lower eyelid fat pads was previously reported as a specific phenotype associated with the *OBSL1* c.458dupG variant (Huang et al., 2021); however, we found that a patient with *CUL7* mutation also had changes in the lower eyelid fat pad. Overall, no specific genotype-phenotype associations have been found in patients with 3M syndrome.

To date, 69 *CUL7* mutations associated with type 1 3M syndrome have been reported in the HGMD database. These mutations span almost the entire *CUL7* coding sequence and inactivate CUL7 through mRNA decay or protein truncation, resulting in the inability of CUL7 protein to recruit ROC1, thus preventing substrate ubiquitination and causing cyclin D1, insulin receptor substrate-1 and other substrates to accumulate, eventually leading to disordered cell proliferation and differentiation in the quiescent zone of the growth plate, resulting in short stature (Isik et al., 2021). Further, *CUL7* deletion leads to dysregulation of cell microtubule dynamics, thus affecting the binding of microtubules to the centromere, leading to defects in mitosis and cell division, indicating that CUL7 protein has a potential function in cell division regulation (Fu et al., 2022). The mitosis and cytokinesis defects caused by loss of CUL7 function may be related to the growth retardation observed in patients with 3M syndrome.

*OBSL1* maps to human chromosome 19q13.2-q13.32 and is a member of the UNC-89/Obscurin gene family. The OBSL protein is a key component of the cytoskeletal network and functions through interactions with myosin proteins, primarily localized to the cell membrane and around the nucleus (Wang et al., 2019). To date, 39 *OBSL1* mutations have been reported as pathogenic causes of type 2 3M syndrome. Further, cells with OBSL1 knocked out have a phenotype consistent with CUL7 knockout, with changes in microtubule dynamics and mitotic dysfunction, and cells dying in polyploid or tetraploid states (Zhang et al., 2019), indicating that the two genes may have important roles in the same pathway involved in growth and development regulation, which could also explain why there is no significant difference in phenotype between patients with mutations in these two genes.

Effective treatment for 3M syndrome is still lacking, and use of growth hormone treatment is controversial. Most early studies reported that rhGH was ineffective for patients with 3M syndrome, and did not recommend growth hormone treatment (Huber et al., 2011; Meazza et al., 2013; Wang et al., 2019; Khachnaoui-Zaafrane et al., 2022); however, there have been literature reports that many patients have been effectively treated with growth hormone in recent years. Kyung Lee et al. (2020) reported two cases of children with 3M syndrome and precocious puberty who were treated with gonadotrophin-releasing hormone agonists combined with growth hormone, both of whom showed clear height catch up and improved adult height. Isik et al. (20221) reported two patients with 3M syndrome accompanied by growth hormone deficiency after treatment with rhGH, both patients showed clear height catch-up. At present, there are only a few case reports on growth hormone treatment of 3M syndrome, no large-scale clinical trials and mata meta-analysis, which will be the direction of our future research. There have been no reports of serious adverse reactions in patients with 3M syndrome after treatment with growth hormone. Further, the two patients treated with rhGH in our center exhibited accelerated growth without adverse reactions. Therefore, we believe that growth hormone treatment can be attempted for patients with 3M syndrome, without contraindication; however, its long-term effectiveness requires further study in a larger sample with long-term follow-up.

## 5 Conclusion

In conclusion, we identified novel truncating mutations of *CUL7* and *OBSL1* in Chinese patients with 3M syndrome. Skeletal manifestations may not be typical in some patients with 3M syndrome, which may be accompanied by early developmental delay. Further studies combined with skeletal surveys and growth curve analysis are needed to determine whether growth hormone treatment is effective or not. Genetic testing should be carried out for children with short stature with specific facial features and bone changes, particularly those with height < –3SD, to facilitate early diagnosis.

## Data Availability

The datasets for this article are not publicly available due to concerns regarding participant/patient anonymity. Requests to access the datasets should be directed to the corresponding author
